# Old Black Water

**Published:** 2005-10-15

**Authors:** Lynne S. Wilcox

In 1927, heavy spring rains and swollen tributaries caused the Mississippi River to flood across its vast plains, eventually covering 16 million acres in seven states (1). Hundreds of people lost their lives, months passed before the waters receded, and years passed before the region recovered (2). Federal, state, and local agencies, including the U.S. Public Health Service (USPHS), responded with resources. The American Red Cross established 138 camps and fed more than 600,000 people during the relief and recovery effort (3).

But the response was insufficient. None of the previous flood control and disease prevention planning had anticipated such a large-scale disaster. The Red Cross was forced to ration food portions, which included the traditional "three-m" diet of the South's rural poor: meat (salt pork), meal (cornmeal), and molasses (1). Fresh produce and milk were not available because vegetable fields and grazing land were still under water, livestock had been lost in the flood, and most of the floodplains had been planted in cotton.

History makes little mention of infectious disease outbreaks in the flood's aftermath, although nursing reports from the Red Cross spoke of distributing tons of quinine and administering thousands of inoculations (4). But there was another health threat of major concern to the relief workers: a disease famous for its symptoms of dermatitis, diarrhea, dementia, and death — a disease that killed up to a third of its victims.

The disease was pellagra. By the time of the Mississippi flood in 1927, Dr Joseph Goldberger of the USPHS had conducted extensive research demonstrating that pellagra was caused by a nutritional deficiency (a lack of niacin, or vitamin B_3_), not an infectious agent. He traveled to the flooded areas to evaluate the extent of the disease, consulting in Arkansas, Louisiana, Mississippi, and Tennessee. Goldberger advised the Red Cross to add brewer's yeast to its food rations. Within weeks, people with pellagra were cured, and new cases of pellagra were prevented (1).

Goldberger went on to demonstrate that the flood conditions had exacerbated already high levels of pellagra along the Mississippi River (1). His completed analysis addressed not only the acute conditions of the flood but the underlying agricultural system that persistently discouraged healthy diets and increased the risks for developing overt disease. Other disasters have demonstrated that disadvantaged populations are often hardest hit by the aftermath of catastrophe. In Goldberger's setting, more than half of people with pellagra were sharecroppers, and sharecroppers were primarily African American (1).

The hurricane seasons of 2004 and 2005 remind us that ameliorating chronic diseases during catastrophic events is critical. At this time in public health history, we understand the acute and enduring actions required to protect people from the ravages of heart disease, diabetes, cancer, and other chronic disorders far better than Goldberger's peers. These damaging effects are still most common among disadvantaged populations, and as we respond to current events we must continue to recognize the social and environmental factors that influence disease outcomes.

*Preventing Chronic Disease* will continue its role in supporting the dialogue between chronic disease researchers and practitioners by examining the effect of disaster on chronic diseases. As part of its responsibility for rapid response, the journal has created a special section: "Chronic Disease in Times of Disaster." The journal welcomes editorials, short reports, data summaries, letters to the editor, resource citations, and other relevant material. The journal will review and post information as soon as possible, independent of its regular publication schedule. Additional information on submissions is available in the special section, and potential contributors are encouraged to contact the journal's editors at PCDeditor@cdc.gov to submit queries about their submissions.

In 2004, *Risk and Insurance* magazine published a list of 10 theoretical disasters that have a probability of occurring at least once in 100 years (5). These include an East Coast Category Four hurricane, a Mississippi River flood, a Chicago terrorist attack, and an earthquake in Los Angeles with a magnitude of 7.0 on the Richter scale. If we begin now, we can work to ensure that people with chronic diseases and disabilities will be better protected when the next disaster strikes.
